# Caregivers’ Health Literacy and Gaps in Children’s Medicaid Enrollment: Findings from the Carolina Oral Health Literacy Study

**DOI:** 10.1371/journal.pone.0110178

**Published:** 2014-10-10

**Authors:** Jessica Y. Lee, Kimon Divaris, Darren A. DeWalt, A. Diane Baker, Ziya Gizlice, R. Gary Rozier, William F. Vann

**Affiliations:** 1 Department of Pediatric Dentistry, School of Dentistry, University of North Carolina at Chapel Hill, Chapel Hill, NC, United States of America; 2 Department of Health Policy and Management, UNC Gillings School of Global Public Health, University of North Carolina at Chapel Hill, Chapel Hill, NC, United States of America; 3 Department of Epidemiology, UNC Gillings School of Global Public Health, University of North Carolina at Chapel Hill, Chapel Hill, NC, United States of America; 4 School of Medicine and Cecil G. Sheps Center for Health Services Research, University of North Carolina at Chapel Hill, Chapel Hill, NC, United States of America; 5 Center for Health Promotion and Disease Prevention, University of North Carolina at Chapel Hill, Chapel Hill, NC, United States of America; University of St Andrews, United Kingdom

## Abstract

**Background and Objectives:**

Recent evidence supports a link between caregivers’ health literacy and their children’s health and use of health services. Disruptions in children’s health insurance coverage have been linked to poor health care and outcomes. We examined young children’s Medicaid enrollment patterns in a well-characterized cohort of child/caregivers dyads and investigated the association of caregivers’ low health literacy with the incidence of enrollment gaps.

**Methods:**

We relied upon Medicaid enrollment data for 1208 children (mean age = 19 months) enrolled in the Carolina Oral Health Literacy project during 2008–09. The median follow-up was 25 months. Health literacy was measured using the Newest Vital Sign (NVS). Analyses relied on descriptive, bivariate, and multivariate methods based on Poisson modeling.

**Findings:**

One-third of children experienced one or more enrollment gaps; most were short in duration (median = 5 months). The risk of gaps was inversely associated with caregivers’ age, with a 2% relative risk decrease for each added year. Low health literacy was associated with a modestly elevated risk increase [Incidence Rate Ratio (IRR) = 1.17 (95% confidence interval (CI) 0.88–1.57)] for enrollment disruptions; however, this estimate was substantially elevated among caregivers with less than a high school education [IRR = 1.52 (95% CI 0.99–2.35); homogeneity p<0.2].

**Conclusions:**

Our findings provide initial support for a possible role of caregivers’ health literacy as a determinant of children’s Medicaid enrollment gaps. Although the association between health literacy and enrollment gaps was not confirmed statistically, we found that it was markedly stronger among caregivers with low educational attainment. This population, as well as young caregivers, may be the most vulnerable to the negative effects of low health literacy.

## Introduction

### Health Insurance Coverage Affects Appropriate Health Care during the First Years of Life

Children’s health insurance coverage is a major determinant of preventive and continuous health care [1]–[3]. Low-income children enrolled in Medicaid are more likely to receive timely preventive care, including well-child visits, compared with their counterparts without insurance coverage [4]–[8]. Nationally representative US data confirm that being uninsured is associated with low levels of adherence to professional recommendations for well-child visits, with the lowest rates found among children eligible but not enrolled in public insurance [9]. This is an important observation because well-child visits and preventive care during the first years of life have demonstrable benefits [10]–[12]. Similarly, continuity of care has been linked to favorable health outcomes, including lower emergency department utilization [13]. These observations are aligned with the concept of a ‘medical home’ which, according to the American Academy of Pediatrics, encompasses the characteristics of pediatric care considered essential for all children” and is defined as a “model of primary care that is accessible, continuous, comprehensive, family-centered, coordinated, compassionate, and culturally effective” [14]–[15].

Current legislation supports the expansion in Medicaid coverage in 2014 [16] and has the potential to benefit a large and vulnerable component of the US population. Insurance coverage is an important enabling factor for health care use overall and its absence leads to under-utilization and unmet medical needs [8]; however, its availability is not sufficient for enrollment or the establishment of a medical home [10]. To affect health outcomes, the expansion of coverage must be met with adequate enrollment.

### To What Extent are Disruptions in Health Insurance Detrimental for Children?

Current evidence demonstrates that disruptions (“gaps”) in coverage are likely to decrease both access to a medical home and the likelihood of seeking routine care for young children [17]–[19]. A 2006 report [20] found that between 18–36% of children in four states had insurance gaps over a period of up to 2 years. Other recent studies confirmed that insurance gaps may be frequent, with 25% of children experiencing a gap in Medicaid enrollment over a 12-month period in Oregon [17], and 14–61% of children in five other states experiencing similar gaps over a 4-year period [21]. Because of its frequency and negative effects, unstable health insurance coverage is a phenomenon that needs further investigation as a risk factor for children’s health [2], [22]–[23].

### What is known about the Correlates of Health Insurance Enrollment Gaps?

State Medicaid programs have a compelling interest in enrolling and maintaining coverage for eligible children but there is a paucity of evidence to inform policy makers on relevant strategies and best practices to accomplish these goals [24]. Although state programs strive to keep enrollment and renewal procedures simple, most reports on enrollment gaps implicate caregiver-related factors. For example, studies by DeVoe et al. [17] and Satchell and Pati [22] relied upon nationally representative data and found that poverty and maternal education were the strongest correlates of enrollment gaps. Gaps may be attributed to changes in family circumstances that render parents and children ineligible; nevertheless, caregivers’ difficulty in navigating the complex administrative processes can also explain why those eligible for public or private insurance are challenged to enroll and maintain enrollment. The challenges of the required forms and paperwork are well-documented [20], [25]–[26]. As one example, recent findings indicate that Medicaid enrollment forms may be excessively demanding to read and comprehend [27]; in fact, most states fail to meet their self-established readability criteria [28].

### The Role of Health Literacy

A recent report [29] identified low health literacy as a risk factor for lack of adult health insurance and outlined the direct implications for the anticipated Medicaid expansion and implementation under the Affordable Care Act (ACA). Several studies have documented an association between caregiver health literacy and child health and services’ use [30]–[33]. Health literacy’s relationship to Medicaid enrollment gaps may offer one potential explanation of the relationship between parental health literacy and child health outcomes. To the best of our knowledge, no data exist to date regarding the possible association between caregivers’ health literacy and gaps in their children’s public health insurance enrollment. Our aims were to 1) describe and characterize gaps in Medicaid enrollment of young, low-income children of English-speaking families in North Carolina, and 2) identify caregiver factors associated with this phenomenon, including health literacy.

## Methods

### Ethics statement

Written consent and HIPPA approval were obtained by all participants and the study was approved by the Biomedical Institutional Review Board at the University of North Carolina-Chapel Hill (approval #07-0837).

### Parent study sampling frame

This investigation utilizes data obtained in the context of the Carolina Oral Health Literacy (COHL) Study, which initially enrolled 1,405 clients of the Special Supplemental Nutrition Program for Women, Infants and Children (WIC) in seven counties in NC: Brunswick, Buncombe, Burke, New Hanover, Orange, Robeson, and Wake [34]. The study’s aim was to investigate the impact of caregivers’ health literacy on their children’s oral health and use of services. The study population consisted of low-income predominantly female caregivers who were 18 years of age or older, English-speaking, and the primary caregiver of a Medicaid-eligible healthy child. Purposeful quota sampling [35] was used to ensure adequate representation of African American (AA; 40%) and American Indian (AI; 20%) participants. Detailed descriptions of the study procedures, interview instruments and baseline outcomes have been reported previously [36]–[38]. In brief, non-random WIC sites were selected based on geographic region, rural or urban makeup, population demographics, existence of very active WIC clinics, and established working relationship with the investigators [34]. Consecutive WIC-attending clients were approached by study personnel while in the waiting areas and asked if they would to 8 questions included in the study’s eligibility screener. Caregivers who were determined to be eligible and agreed to participate were accompanied to a private area to complete the in-person study interview. Of note, to qualify for WIC, caregivers must have an income <185 percent of the federal poverty level.

### Data Sources

Data were collected in 30-minute in-person interviews with one of the two trained study interviewers between July 2008-July 2009 in the domains of socio-demography, caregivers’ self-reported personal and children’s general and dental health status, dental knowledge and behaviors, quality of life, dental neglect, general self-efficacy, and health literacy. Study personnel screened eligible participants to ensure that unique children were enrolled in the cohort. In June 2011, we obtained Medicaid enrollment and claims files for children from the NC Division of Medical Assistance, ranging from January 2004 to December 2010, a period that covered the lifetime Medicaid enrollment history for all children up to the end of CY-2010. We merged these files with interview data that were obtained at baseline using the children’s unique Medicaid identification number that was provided by the caregivers at study enrollment. All COHL children were Medicaid-eligible or enrolled at baseline, as per our inclusion requirements. Of the 1405 caregivers recruited, 1242 had children with a Medicaid identification number at the baseline interview. Children’s unique Medicaid identification number was used to link interview and Medicaid claims data. Subsequently, we excluded dyads wherein children had no Medicaid data (n = 31; 2% of total), caregivers under 18 years old (n = 2; 0.2%), and one caregiver (0.1%) who had missing information on health literacy. Thus, the final analytical sample included 1208 child/caregiver dyads.

### Outcome Definition and Covariates

The study’s primary analytical endpoint was the incidence of Medicaid enrollment disruptions or “gaps”. During the study period (2004–2010) Medicaid eligibility criteria in NC, as well as procedures of enrollment and re-enrollment varied for different categories of child/caregiver dyads; however, these criteria included, among others, income, resources, residence, and proof of citizenship. Re-enrollment processes included periodic verifications of eligibility criteria at intervals ranging between 3–12 months, unless changes were self-reported by the beneficiaries. The current list of eligibility criteria and required documentation can be found at: http://www.ncdhhs.gov/dma/forms/famchld.pdf. For the purposes of this study we defined gap as a period during which a child was Medicaid-eligible but not enrolled, which was preceded *and* followed by periods of enrollment, as described by Copper et al. [39]. This definition of enrollment disruption was conservative, in a manner that prevented children with a permanent change in Medicaid-eligibility from being classified as having a gap. In such instances, there would be no subsequent re-enrollment in the NC files and the child would be labeled “lost to follow-up” rather than having an enrollment gap.

Apart from caregivers’ difficulty with necessary forms and processes and administrative reasons, there are several other reasons why enrollment interruptions occur for children covered by Medicaid. Among others, these include: a child/family moving out of state, a child’s acquisition of comprehensive private health insurance, a caregiver’s request for termination of coverage, and family income changes. Any of these result in permanent eligibility changes without subsequent re-enrollment. We cannot ascertain with certainty the reason for permanent eligibility changes and some may be due to difficulty in processes/procedures; however, our goal for using a conservative definition of gaps was to ensure we were capturing administrative-related lapses due to parental actions that resulted in an interruption in enrollment.

Enumeration of enrolled/non-enrolled months began at the month of the baseline COHL interview and ended in December 2010. For each child, we identified and counted enrolled/non-enrolled periods and calculated their duration in months. We calculated ‘enrollment proportion’ as the ratio of months enrolled divided by months eligible with a range of 0–100. History of Medicaid enrollment was available from January 2004-December 2010, a period that included all available Medicaid data up to 2010 for all children. To further characterize patterns of Medicaid enrollment, we calculated periods of non-enrollment, enrollment gaps, and percent time enrolled both during the study duration and the entire period available.

We measured health literacy using a comprehension and numeracy-based instrument, the Newest Vital Sign (NVS). The NVS is a nutritional label accompanied by six questions, requiring approximately three minutes for completion. The NVS is a validated test with good psychometric properties and it has been used extensively since its introduction [40]–[41]. According to Weiss et al. [40] who developed and tested the NVS, the instrument is reliable (Cronbach alpha >0.76 in English), correlates with the test of functional health literacy in adults (TOFHLA), requires 3 minutes for administration and, thus, is suitable for use as a quick screening test for limited health literacy. According to Osborn et al. [42], the NVS is able to reliably identify individuals with limited literacy skills, which was the group of interest in this study (vs. the upper end of the literacy spectrum). In the context of our study, the NVS’s Cronbach’s *alpha* was 0.75. The NVS score ranges from 0–6, wherein values of 0–1 are indicative of “low”, 2–3 “moderate” and 4–6 “higher health literacy” [42]. We used this 3-level categorical classification as well as a dichotomous one where NVS score 0–1: “low health literacy” and 2–6: “higher health literacy”. The examination of the latter dichotomy was driven by our working hypothesis which considered low caregiver health literacy as a predictor of children’s Medicaid enrollment gap; in this scenario, a contrast between the lowest category of the NVS index (containing individuals most likely to face difficulties with forms and administrative processes) and higher ones was the most meaningful one.

Socio-demographic covariates included caregivers’ race, sex, age, education, marital status, number of children, and child’s age. Race was self-reported and categorized as white, African American (AA), and American Indian (AI). Caregivers’ age was measured in years and classified as a tertile-categorical variable for descriptive purposes and a continuous variable for analytical purposes. Education was grouped in four categories: less than high school (HS) education, HS/General Education Diploma (GED), some technical or college training, and college or higher education. Marital status was classified as single, married, and divorced/separated/other. Children’s age was measured in months and was categorized in five groups: 0–11, 12–23, 24–25, 36–47, and 48–59 months.

As a measure of caregivers’ self-perceived health status, we used the National Health and Nutrition Examination Study (NHANES) self-reported item containing five possible answers: excellent, very good, good, fair, and poor. For analytical purposes, the responses were converted to a dichotomous variable, excellent/very good/good *versus* fair/poor.

### Data availability

We have created and made available a de-identified dataset to accompany this manuscript, as supplemental material available online. This tab-delimited dataset (Dataset S1) contains all original and derived variables described and used for this study’s analyses, including a data dictionary as a separate spreadsheet.

### Statistical Analyses

We used descriptive statistics for our initial data presentation and χ^2^ tests to compare the distribution of low/adequate health literacy and Medicaid enrollment gaps across strata of covariates. Because the occurrence of enrollment gaps is a time-dependent event, ignoring the time at-risk could introduce bias of substantial magnitude and unknown direction. For this reason, we used multivariate Poisson regression models that accounted for time at risk using months enrolled as the exposure and a log-link function. Although our modeling approach did not consider the length of each gap, we support that it is superior to the enumeration of months not enrolled due to reasons outlined above. To identify a final model, we employed a backward hierarchical variable selection strategy [43], starting with a full model that included all covariates and a p<0.2 criterion. We included caregivers’ race, age, and general health status *a priori* in all models and obtained exponentiated model coefficients corresponding to Incidence Rate Ratios (IRR) and 95% confidence intervals (CI). For the interpretation of results, we followed an effect-estimation (estimation of the magnitude and precision of the association) *versus* hypothesis-testing (determination of the presence of an association or not) approach [44].

For sensitivity analysis, we developed a second identical model considering all Medicaid enrollment history available, prior to and after enrollment in the COHL study. All Medicaid enrollment history covered the lifetime period for all enrolled children, up to December 2010. In addition, to explore for potential vulnerable sub-groups, we conducted a *post hoc* homogeneity analysis to determine whether educational attainment and race modified the association between caregivers’ health literacy and insurance gaps. To do so, we compared education level and race-specific estimates against the main effect estimate using a homogeneity χ^2^ test. Because these *post hoc* stratified analyses are *de facto* underpowered, we used an a priori p value criterion of <0.2 for the detection of heterogeneity [45]. We used Stata version 12.1 (StataCorp LP, College Station, TX) and SAS version 9.3 (SAS Institute, Cary, NC) software for data analyses.

## Results

The primary caregivers’ socio-demographic characteristics and responses to the self-reported health status items, both overall and stratified by health literacy group, are presented in Table 1. There was a 2∶2∶1 distribution of white, AA, and AI participants with the vast majority of all participants being female. Caregivers’ and children’s mean age at the baseline interview was 27 years and 17 months, respectively. A small proportion (11%) of caregivers reported their general health as fair or poor. Fifteen percent of participants were classified as low health literacy based on their NVS score. This proportion was higher among AA and AI participants and among those who were younger, had lower educational attainment, and worse self-reported health status.

**Table 1:** Distribution of Health Literacy Estimates (NVS) across Levels of Caregivers’ Characteristics, in the COHL Study Follow-up Cohort, North Carolina, 2008–2010.

		Health Literacy (NVS)				
	No.^a^ (%) or	NVS score	Low	Moderate	Higher	?^2^; p
	mean (median)	(95% CI)	n (row %)	n (row %)	n (row %)	
All	1208	3.1 (3.0, 3.2)	199 (16)	508 (42)	501 (41)	
**Race**						<0.001
White	484 (40)	3.8 (3.6, 3.9)	45 (9)	162 (33)	277 (57)	
African American	478 (40)	2.7 (2.6, 2.9)	96 (20)	239 (50)	143 (30)	
American Indian	236 (20)	2.8 (2.6, 3.0)	51 (22)	106 (45)	79 (33)	
**Caregiver’s sex**						0.6
Male	46 (4)	3.3 (2.7, 3.8)	9 (20)	16 (35)	21 (46)	
Female	1162 (96)	3.1 (3.0, 3.2)	190 (16)	492 (42)	480 (41)	
**Caregiver’s age**(years, tertiles; range)						<0.001
* Q1* (18.0, 23.0)	20.8 (20.9)	2.8 (2.7, 3.0)	71 (18)	202 (50)	130 (32)	
* Q2* (23.1, 28.4)	25.5 (25.3)	3.4 (3.3, 3.6)	51 (13)	155 (38)	197 (49)	
* Q3* (28.5, 65.6)	35.0 (33.2)	3.2 (3.0, 3.4)	77 (19)	151 (38)	174 (43)	
**Education**						<0.001
Did not finish high school	288 (24)	2.2 (2.1, 2.4)	87 (30)	143 (50)	58 (20)	
High school diploma or GED	467 (39)	3.0 (2.8, 3.1)	80 (17)	212 (45)	175 (37)	
Some technical/college or higher	453 (37)	3.9 (3.8, 4.1)	32 (7)	153 (34)	268 (59)	
**Marital status**						<0.001
Single	772 (64)	2.9 (2.7, 3.0)	149 (19)	363 (47)	260 (34)	
Married	316 (26)	3.7 (3.5, 3.9)	33 (10)	103 (33)	180 (57)	
Divorced/separated/other	120 (10)	3.5 (3.2, 3.8)	17 (14)	42 (35)	61 (51)	
**Number of children**						0.6
1	479 (40)	3.2 (3.1, 3.4)	73 (15)	204 (43)	202 (42)	
2	395 (33)	3.1 (3.0, 3.3)	62 (16)	173 (44)	160 (41)	
3	196 (16)	3.2 (2.9, 3.4)	34 (17)	76 (39)	86 (44)	
≥4	136 (11)	2.9 (2.6, 3.2)	29 (21)	55 (40)	52 (38)	
**Caregiver general health**						0.4
Excellent/Very good/Good	1076 (89)	3.2 (3.1, 3.3)	173 (16)	451 (42)	452 (42)	
Fair/Poor	132 (11)	2.9 (2.6, 3.2)	26 (20)	57 (43)	49 (37)	

*Note*. NVS = Newest Vital Sign; COHL = Carolina Oral Health Literacy; CI = Confidence Interval; HS = High School; GED = General Educational Development. The sample size was n = 1208.

a Column figures may not add up to total because of missing values.

The median follow-up time was 25 months (mean = 24; range = 18–29), with 11% of children experiencing an interruption of Medicaid enrollment with no subsequent re-enrollment. This latter phenomenon was almost twice as high among children of caregivers with some technical/college or higher education *versus* those with less than high school education (15% *versus* 8%). Thirty percent of children experienced at least one enrollment gap (Table 2). Relative to gaps, the majority of the children (90%) experienced only one, 9% had two, and 1% had three. Twenty percent of these interruptions had duration of one month, 50% four months or less, while 21% were 12 months or longer. As anticipated, children with no gaps were enrolled longer, on average for six more months compared to those with gaps. Relative to bivariate associations, continuous enrollment was more frequent among children of older caregivers, older children, and those with more siblings (Table 2). Continuous enrollment was more frequent among children whose caregivers reported having better health but the difference was small (3%).

**Table 2:** Medicaid Enrollment Estimates (Children with and without Gaps in Enrollment) by Caregiver Characteristics among the COHL Study Follow-up Cohort during the Follow-up Period (Median 25 Months), North Carolina, 2008–2010.

	No enrollment gap^a^				One or more enrollment gaps^a^			
		Enrollment (months)				Enrollment (months)		
	No.^b^(row %)	Mean	Median(range)	?^ 2^; p^c^	No.^b^(row %)	Mean	Median(range)	EnrollmentProportion^d^ (95% CI)
Total	846 (70)	24	25 (8–30)		362 (30)	18	19 (1–29)	0.71 (0.68–0.74)
**Race**				0.2				
White	329 (68)	25	25 (8–30)		155 (32)	18	19 (2–29)	0.71 (0.66–0.75)
African American	334 (70)	25	27 (18–30)		144 (30)	18	19 (1–29)	0.71 (0.66–0.75)
American Indian	177 (75)	22	21 (11–29)		579 (25)	17	18 (4–29)	0.76 (0.70–0.81)
**Caregiver’s sex**				.02				
Male	25 (54)	26	27 (18–30)		21 (46)	17	17 (3–29)	0.64 (0.51–0.78)
Female	821 (71)	24	25 (8–30)		341 (29)	18	19 (1–29)	0.71 (0.69–0.74)
**Caregiver’s age** (tertiles)				0.03				
* Q1*	264 (66)	24	24 (18–30)		139 (34)	19	20 (3–29)	0.76 (0.72–0.80)
* Q2*	284 (70)	24	25 (8–30)		119 (30)	17	18 (3–29)	0.70 (0.65–0.75)
* Q3*	298 (74)	25	25 (18–30)		104 (26)	17	18 (1–29)	0.66 (0.60–0.71)
**Child’s age** (months; atbaseline interview)				0.05				
0–11	347 (66)	25	25 (18–30)		182 (34)	17	18 (2–28)	0.69 (0.65–0.73)
12–23	177 (72)	25	25 (11–30)		70 (28)	19	20 (1–29)	0.75 (0.69–0.81)
24–35	154 (74)	24	23 (8–30)		55 (26)	18	20 (3–29)	0.74 (0.68–0.81)
36–47	146 (75)	24	25 (18–30)		48 (25)	17	17 (3–28)	0.69 (0.62–0.76)
48–59	22 (76)	21	19 (18–30)		7 (24)	16	17 (7–25)	0.73 (0.50–0.95)
**Education**				0.8				
<HS	204 (71)	24	25 (8–30)		84 (29)	19	21 (3–29)	0.79 (0.74–0.84)
HS/GED	330 (71)	24	25 (18–30)		137 (29)	18	19 (3–29)	0.72 (0.68–0.77)
Some college/technical or more	312 (69)	25	25 (18–30)		141 (31)	16	18 (1–29)	0.65 (0.61–0.70)
**Marital status**				0.5				
Single	537 (70)	24	25 (8–30)		235 (30)	19	20 (2–29)	0.75 (0.72–0.78)
Married	221 (70)	24	25 (18–30)		95 (30)	16	16 (2–29)	0.63 (0.57–0.68)
Divorced/separated/other	88 (73)	24	25 (18–30)		32 (27)	16	17.5 (1–26)	0.67 (0.56–0.78)
**Number of children**				0.03				
1	313 (65)	24	24 (18–30)		166 (35)	18	19 (2–29)	0.71 (0.67–0.75)
2	294 (74)	25	25 (18–30)		101 (26)	18	18 (1–29)	0.72 (0.67–0.77)
3	139 (71)	24	25 (11–30)		57 (29)	17	19 (3–29)	0.69 (0.62–0.76)
≥4	98 (72)	25	26 (8–30)		38 (28)	19	19.5 (6–29)	0.74 (0.65–0.82)
**Caregiver** **general health**				0.4				
Excellent/Very good/Good	758 (70)	24	25 (8–30)		318 (30)	18	19 (1–29)	0.71 (0.69–0.74)
Fair/Poor	88 (67)	25	25 (18–30)		44 (33)	17	17.5 (2–29)	0.69 (0.61–0.77)
**Health Literacy** (NVS)				0.8				
Low^e^	137 (69)	24	24 (8–30)		62 (31)	19	19 (4–29)	0.77 (0.72–0.83)
Moderate^f^	360 (71)	24	25 (18–30)		148 (29)	18	19 (1–29)	0.71 (0.67–0.75)
Higher^g^	349 (70)	25	25 (11–30)		152 (30)	17	18 (2–29)	0.69 (0.65–0.73)

*Note*. COHL = Carolina Oral Health Literacy; CI = Confidence Interval; HS = High School; GED = General Educational Development; NVS = Newest Vital Sign. The sample size was n = 1208.

a Enrollment gap was defined as any disruption in Medicaid enrollment within a period of eligibility, preceded and superseded by periods of enrollment.

b Column figures may not add up to total because of missing values.

c Corresponds to X^2^ tests of the equality of distribution of participants with and without enrollment gap across categories of caregiver characteristics.

d Enrollment proportion was calculated for children with enrollment gaps as the ratio of months enrolled over months eligible.

e Defined as NVS score: 0–1.

f Defined as NVS score: 2–3.

g Defined as NVS score: 4–6.

We present the final Poisson regression model (A) for enrollment gap incidence in Table 3. After adjusting for race, education, gender, and all other covariates, inclusion in the low health literacy group was associated with a modestly elevated risk for an enrollment gap (adjusted IRR = 1.17; 95% CI 0.89–1.54). The risk of gaps was inversely associated with caregivers’ age, with a 2% relative risk decrease for each added year. An association between female gender and decreased risk of enrollment gaps was also noted; however, this finding was based on an analysis including only 46 male caregivers and thus should be viewed with caution. Stratified analyses (Table 4) revealed that educational attainment significantly modified the association between literacy and gaps (homogeneity p<0.2), with the highest risk estimates found among caregivers with less than high school education (IRR = 1.52; 95% CI 0.99–2.35). Race-stratified estimates did not depart substantially from homogeneity. Inspection of the exploratory models (B) that considered all available Medicaid enrollment history did not reveal any important differences compared to our main analyses.

**Table 3:** Results of Multivariate Poisson Regression Modeling of Medicaid Enrollment Gap Incidence on Caregiver and Child Characteristics, among the COHL Study Follow-up Cohort in North Carolina, during the Study Period (A) and Using all Available Medicaid History (B) between January 2004 and December 2010.

	Model A^a^:			Model B^a^:		
	COHL study follow-up period			All available Medicaid history		
	IRR	95% CI	p	IRR	95% CI	p
**Health literacy** (NVS)						
Low^b^	1.17	0.89–1.54	0.3	1.10	0.87–1.38	0.4
Moderate-Higher^c^	1.00	*referent*		1.00	*referent*	
**Race**						
White	1.00	*referent*		1.00	*referent*	
African American	0.91	0.73–1.13	0.4	0.93	0.77–1.12	0.4
American Indian	0.86	0.64–1.15	0.3	0.94	0.75–1.18	0.6
**Education** (ordinal categorical)	1.10	0.96–1.26	0.2	1.08	0.96–1.21	0.2
**Caregiver’s sex**						
Male	1.00	*referent*		1.00	*referent*	
Female	0.53	0.35–0.80	0.003	0.62	0.43–0.90	0.01
**Caregiver’s age** (years; continuous)	0.98	0.96–1.00	0.01	0.98	0.96–0.99	<0.001
**Child’s age** (years; ordinal categorical)	0.93	0.86–1.02	0.1	0.99	0.93–1.06	0.9
**Caregiver general health**						
Excellent/Very good/Good	0.83	0.61–1.12	0.2	0.88	0.69–1.14	0.3
Fair/Poor	1.00	*referent*		1.00	*referent*	

*Note*. NVS = Newest Vital Sign; COHL = Carolina Oral Health Literacy; IRR = Incidence Rate Ratio; CI = Confidence Interval. The sample size was n = 1208.

a Variable selection was based on a backward hierarchical procedure starting from a full model and a *P*<.2 criterion; caregivers’ race, age and general health were included a priori in the models; time at risk for a gap was considered time (months) of Medicaid enrollment.

b Defined as NVS score: 0–1.

c Defined as NVS score: 2–6.

**Table 4:** Estimates of the Association between Caregivers’ Low Health Literacy (Defined as NVS Score of less than 2) and the Incidence of Children’s Medicaid Enrollment Gaps, Overall and Stratified by Educational Attainment, among the COHL Study Follow-up Cohort in North Carolina, during the Study Period (A) and Using all Available Medicaid History (B) between January 2004 and December 2010.

	Model A^a^:		Model B^a^:		
	COHL study follow-up period		All available Medicaid history		
	IRR	95% CI	IRR	95% CI	
**All**	1.17	0.89–1.54	1.10	0.86–1.40	
**Race**					
Whites	1.23	0.73–2.06	1.29	0.85–1.96	
African American	1.14	0.76–1.69	1.05	0.75–1.48	
American Indian	1.18	0.64–2.16	0.96	0.58–1.57	
Homogeneity (χ^2^)^b^ *P*	0.97		0.63		
**Education**					
Did not finish high school	1.52	0.99–2.35	1.36		0.97–1.92
High school diploma or GED	1.07	0.68–1.69	1.07		0.73–1.58
Some technical/college training or higher	0.67	0.31–1.45	0.65		0.34–1.24
Homogeneity (χ^2^)^b^ *P*	0.17		0.13		

*Note*. NVS = Newest Vital Sign; COHL = Carolina Oral Health Literacy; IRR = Incidence Rate Ratio; CI = Confidence Interval; GED = General Educational Development. The sample size was n = 1208.

a Variable selection was based on a backward hierarchical procedure starting from a full model and a *P*<.2 criterion; caregivers’ race, age and general health were included a priori in the models; time at risk for a gap was considered time (months) of Medicaid enrollment.

b Wald χ^2^ test of a common IRR (across-strata homogeneity).

## Discussion

In this study of approximately 1200 young children of low-income predominantly female caregivers enrolled in WIC, 30% had a gap in Medicaid enrollment over 25 months. Most experienced only one gap of short duration. Our findings revealed a weak association between caregivers’ health literacy and their children’s Medicaid enrollment gaps; in the entire cohort, independent of race, education, and age, low health literacy was associated with a non-significant gap risk increase of about 20%. Nevertheless, this effect was substantially elevated among caregivers with less than high school education compared to those with high school or higher education; caregivers with low education may be the most vulnerable to the effects of low health literacy. Moreover, above and beyond other caregiver factors, caregivers’ age was inversely associated with the risk of gaps.

The concept of Medicaid’s serving as a revolving-door for some children has been recognized and more steps must be taken by state Medicaid programs to alleviate this problem. While we cannot confirm it using our data, this phenomenon may be more closely related to administrative issues (i.e. difficulty with the enrollment and maintenance process) rather than eligibility disruptions. States are required to record and report on children’s coverage stability but this has generally not been undertaken [46]. It is not uncommon for children who are uninsured for less than a full year to be reported as insured and this cohort of children has received less attention in public health research and policy discussions [23]. Olson [2] found that children insured for part of the previous year had higher proportions of delayed care and unmet medical care *versus* those uninsured for the full year. A similar finding was reported by DeVoe and colleagues [17], who compared children with gaps longer than six months *versus* those never insured.

Children from poor and near-poor families have been reported to be 4–5 times more likely to have lapsed coverage than those from high-income families [22]. At the same time, decreased preventive care utilization among children who had Medicaid insurance gaps was irrespective of their insurance status after discontinued enrollment [47]. Taken together, these findings underscore the dramatic effects that interruptions may have for children, their families, and the health care system. The association between Medicaid interruptions and subsequent children’s health outcomes and related costs is fertile ground for future research.

As more health literacy findings are reported, the multi-level effects of health literacy are becoming increasingly evident. Health literacy is recognized already as having a pivotal role in numerous health outcomes [48]–[49], insurance status [29], and health disparities [50]–[51], and recently was termed the “sixth vital sign” [52]. Strategies that attempt to circumvent or mitigate the effects of low health literacy, such as the Federal Plain Language Act and simplification of enrollment procedures are promising [53], but more steps towards implementation are necessary. Although it is impossible to quantify the precise contributors to the relationship between health literacy and Medicaid enrollment gaps from our data, it appears that the readability of Medicaid forms [27] and user-friendliness of the enrollment renewal process [26], [54]–[55] are potential targets for addressing literacy-sensitive concerns. This notion was recently mirrored by findings reported by Pati et al. [56] who found that mothers with inadequate health literacy were less likely to receive child care subsidy than those with adequate health literacy, implicating the application processes as health literacy-sensitive step.

Our findings on the incidence of enrollment gaps are consistent with previous reports of Medicaid insurance gaps among young children. Previously published estimates [20], [46], [57] of any gap of enrollment were 25% over a 12-month period in Oregon, 26–35% over 18 months in Ohio, 18% over a 2-year period in Louisiana, 25% over 12 months in Rhode Island, and 33% over an 18-month period in in Virginia. State, community, and local population-specific parameters may account for these relatively small variations. Our finding of increased risk for children’s enrollment gaps among younger caregivers is a likely reflection of additional stressors and strains experienced by young parents/caregivers compared to older ones that could include education, employment, transportation, or others.

The finding of a weak association between enrollment gaps and literacy in the full cohort analysis requires further investigation. First, it must be acknowledged that in absence of statistical confirmation, an association may in fact be non-existent. Further, it is possible that the negative effects of low health literacy are manifested only among vulnerable subsets of the population, including those with low educational attainment, a scenario supported by our data. It is also possible that truly a weak association exists; this result might be a reflection of the NC Medicaid Program’s success in addressing issues with the enrollment and maintenance processes and caregiver community support (i.e. an effect of WIC and practice health coordinators). In fact, while NC had the highest readability level goal (6–8 grade) for enrollment forms among all states with such guidelines, it was 40^th^ in terms of actual readability level [28]. This parallels our finding of a 52% risk increase for enrollment gaps among caregivers in the lowest educational attainment group. Nevertheless, other differences in caregivers’ age, family conditions, or children’s health care needs may influence the likelihood to enroll in Medicaid and remain continuously enrolled. As an example, Cooper et al. [39] found that among children with asthma, those without insurance gaps had more hospitalizations compared with those with insurance gaps. This finding was a possible consequence of health problem-initiated and enrollment pattern driven care-seeking behavior.

Evidence supporting an association between parental health literacy and child health insurance status has been reported by large, rigorous studies, including those by Pati et al. [56] and Yin et al. [58]. Although these studies did not investigate enrollment gaps, they add to the growing evidence base linking health literacy with children’s insurance status. The findings of the DeVoe et al. [17] and Satchell and Pati [22] studies underscored the importance of socio-economic stressors, including poverty and maternal education as strong predictors of enrollment gaps. This effect was reflected in our study’s stratified analyses, wherein the effect of low health literacy varied significantly according to education level, with the largest estimates found among the lowest-educated caregivers.

Our findings have limitations. This WIC study cohort was not a probability sample, an important factor because WIC enrollment alone has been shown to confer benefits for child health behaviors and outcomes [59]–[60]. The WIC program is the largest public health nutrition program in the US and, as such, serves a large percentage of low-income children and their mothers. To put this in perspective, in fiscal year 2013, more than 2 million women and infants nationwide participated in WIC, whereas there were 264,000 WIC participants in North Carolina. Our sample size was modestly sized, including approximately 1,200 dyads and 199 caregivers with low health literacy; however, it must be noted to the best of our knowledge no other cohorts to-date exist with person-level data on parents’ health literacy and children’s Medicaid enrollment information. For many practical reasons, our population was limited to English-speaking caregivers over the age of 18, limiting the generalizability of our findings beyond that demographic group. Although there is a Spanish version of the NVS tool, most of our survey instruments at the time of the study had not been developed for or validated in Spanish. Because Hispanic children are often the most likely to have insurance gaps [22], the absence of this demographic group is an obvious study limitation. Future studies among these high-risk, vulnerable groups are warranted. Another limitation that must be pointed out is that NVS is only one of many available instruments available to assess health literacy; as a comprehension and numeracy-based test, it is not a comprehensive health literacy assessment.

Understanding Medicaid enrollment gaps is a complex issue. State differences in the enrollment and re-enrollment processes warrant the investigation of the association between health literacy and enrollment gaps in diverse population-based samples in different states. It is possible that enrollment and re-enrollment in Medicaid require cognitive and/or executive functioning skills not well-captured by the instrument used in this study. Prolonged interruptions could be due to changes in eligibility, but gaps can be due to administrative issues on both the program and the client’s sides. Our data included rich information from the perspective of the client, but lacked information from the perspective of the program.

### Summary and implications

Health insurance gaps have been linked to increased health disparities [61], leading to a poorer quality of health care [62]. Accordingly, correcting problems with children’s health insurance enrollment and coverage should be a priority for stakeholders. We found that 30% of young children experienced one or more enrollment gaps over a 2-year period. Our data did not reveal any important association between caregivers’ health literacy and the incidence of these gaps; however, they indicated that low health literacy may be a risk factor for enrollment gaps among the lowest-educated caregivers, who may be the most vulnerable to its detrimental effects. Moreover, caregivers’ young age showed an association with increased risk for enrollment gaps. Considering the economy of scale of the anticipated health insurance coverage expansion in 2014, insurance coverage and continuity issues are certain to become ever more important challenges. Because health literacy issues are, unlike many other social determinants, amenable to interventions, it is critical that they are kept on the agendas of policy makers. Although age is a non-intervenable factor, it may be pertinent to consider caregivers’ (young) age as a possible additional risk factor for their children’s public insurance enrollment disruptions.

The Congressional Budget Office projects that the currently 34 million people that are covered by Medicaid will increase to 47 million in 2014 with the implementation of the ACA [63]. Considering this huge expansion, health insurance coverage should be examined from a social and behavioral perspective to inform and guide states’ actions in the implementation of the Medicaid coverage expansion [16], [64]–[66]. Gaps in insurance coverage should be considered with a rigor similar to non-enrollment. More research is needed to comprehensively characterize health system and individual-level risk factors for public health insurance gaps; however, it must be acknowledged that population-wide, policy solutions such as continuous eligibility and automatic re-enrollment in public insurance may be more effective approaches compared to “individual risk factor” strategies. Beyond the obvious utility of identifying susceptible groups or individuals (i.e., young caregivers of children), it is critical that planned interventions are evidence-based with attention to the critical issues of health literacy and cultural appropriateness.

## Supporting Information

Dataset S1 **Tab-delimited dataset containing all original and derived variables described and used for this study’s analyses, including a data dictionary as a separate spreadsheet.**
(ZIP)

## References

[1] Chung PJ, Lee TC, Morrison JL, Schuster MA (2006) Preventive care for children in the United States: quality and barriers. Annu Rev Public Health 27: 491–515. 16533127 10.1146/annurev.publhealth.27.021405.102155

[2] Olson LM, Tang SF, Newacheck PW (2005) Children in the United States with discontinuous health insurance coverage. N Engl J Med 353: 382–291. 16049210 10.1056/NEJMsa043878

[3] Newacheck PW, Stoddard JJ, Hughes DC, Pearl M (1998) Health insurance and access to primary care for children. N Engl J Med 338: 513–519. 9468469 10.1056/NEJM199802193380806

[4] Fisher MA, Mascarenhas AK (2009) A comparison of medical and dental outcomes for Medicaid-insured and uninsured Medicaid-eligible children: a U.S. population-based study. J Am Dent Assoc 140: 1403–1412. 19884400 10.14219/jada.archive.2009.0078

[5] Fisher MA, Mascarenhas AK (2007) Does Medicaid improve utilization of medical and dental services and health outcomes for Medicaid-eligible children in the United States? Community Dent Oral Epidemiol 35: 263–271. 17615013 10.1111/j.1600-0528.2007.00341.x

[6] Freed GL, Clark SJ, Pathman DE, Schectman R (1999) Influences on the receipt of well-child visits in the first two years of life. Pediatrics 103: 864–869. 10103323

[7] St Peter RF, Newacheck PW, Halfon N (1992) Access to care for poor children. Separate and unequal? JAMA 267: 2760–2764. 1578595

[8] Newacheck PW, Halfon N (1988) Preventive care use by school-aged children: differences by socioeconomic status. Pediatrics 82: 462–468. 3043369

[9] Selden TM (2006) Compliance with well-child visit recommendations: evidence from the Medical Expenditure Panel Survey, 2000–2002. Pediatrics 118: e1766–1778. 17142499 10.1542/peds.2006-0286

[10] Starfield B, Shi L (2004) The medical home, access to care, and insurance: a review of evidence. Pediatrics 113: 1493–1498. 15121917

[11] Savage MF, Lee JY, Kotch JB, Vann WF Jr (2004) Early preventive dental visits: effects on subsequent utilization and costs. Pediatrics 114: e418–423. 15466066 10.1542/peds.2003-0469-F

[12] Hakim RB, Bye BV (2001) Effectiveness of compliance with pediatric preventive care guidelines among Medicaid beneficiaries. Pediatrics 108: 90–97. 11433059 10.1542/peds.108.1.90

[13] Christakis DA, Wright JA, Koepsell TD, Emerson S, Connell FA (1999) Is greater continuity of care associated with less emergency department utilization? Pediatrics 103: 738–742. 10103295 10.1542/peds.103.4.738

[14] Strickland BB, Jones JR, Ghandour RM, Kogan MD, Newacheck PW (2011) The medical home: health care access and impact for children and youth in the United States. Pediatrics 127: 604–611. 21402643 10.1542/peds.2009-3555

[15] National Association of Pediatric Nurse Practitioners (2003) NAPNAP position statement on credentialing and privileging for pediatric nurse practitioners. J Pediatr Health Care 17: A22–23. 14576637 10.1016/s0891-5245(03)00155-x

[16] Landers RM (2012) The Dénouement of the Supreme Court’s ACA Drama. N Engl J Med 367: 198–199. 22747179 10.1056/NEJMp1206847

[17] DeVoe JE, Graham A, Krois L, Smith J, Fairbrother GL (2008) “Mind the Gap” in children’s health insurance coverage: does the length of a child’s coverage gap matter? Ambul Pediatr 8: 129–134. 18355742 10.1016/j.ambp.2007.10.003 PMC4918900

[18] Federico SG, Steiner JF, Beaty B, Crane L, Kempe A (2007) Disruptions in insurance coverage: patterns and relationship to health care access, unmet need, and utilization before enrollment in the State Children’s Health Insurance Program. Pediatrics 120: e1009–1016. 17908722 10.1542/peds.2006-3094

[19] Wise PH, Wampler NS, Chavkin W, Romero D (2002) Chronic illness among poor children enrolled in the temporary assistance for needy families program. Am J Public Health 92: 1458–1461. 12197973 10.2105/ajph.92.9.1458 PMC1447258

[20] Summer L, Mann C (2006) Instability of public health insurance coverage for children and their families: causes, consequences, and remedies. New York: The common wealth fund. Available: http://www.commonwealthfund.org/~/media/Files/Publications/Fund%20Report/2006/Jun/Instability%20of%20Public%20Health%20Insurance%20Coverage%20for%20Children%20and%20Their%20Families%20%20Causes%20%20Consequence/Summer_instabilitypubhltinschildren_935%20pdf.pdf. Accessed 2014 Sept 19.

[21] Fairbrother GL, Emerson HP, Partridge L (2007) How stable is medicaid coverage for children? Health Aff (Millwood) 26: 520–528. 17339682 10.1377/hlthaff.26.2.520

[22] Satchell M, Pati S (2005) Insurance gaps among vulnerable children in the United States, 1999–2001. Pediatrics 116: 1155–1161. 16264003 10.1542/peds.2004-2403 PMC1370262

[23] Tang SF, Olson LM, Yudkowsky BK (2003) Uninsured children: how we count matters. Pediatrics 112: e168–173. 12897324 10.1542/peds.112.2.e168

[24] Wachino V, Weiss A (2009) Maximizing kids’ enrollment in Medicaid and SCHIP: What works in reaching, enrolling, and retaining eligible children. National Academy for State Health Policy, Washington, DC. Available: http://nashp.org/sites/default/files/Max_Enroll_Report_FINAL.pdf?q=files/Max_Enroll_Report_FINAL.pdf. Accessed 2014 Sept 19.

[25] Price J, Boswell J, Lessard M, Wood K (2006) Why Parents Disenroll Children from Public Health Insurance: The Case of Southeastern North Carolina. Journal of Applied Social Science 21: 92–106.

[26] DeVoe JE, Westfall N, Crocker S, Eigner D, Selph S, et al (2012) Why do some eligible families forego public insurance for their children? A qualitative analysis. Fam Med 44: 39–46. 22241340 PMC4407493

[27] Wilson JM, Wallace LS, DeVoe JE (2009) Are state Medicaid application enrollment forms readable? J Health Care Poor Underserved 20: 423–431. 19395839 10.1353/hpu.0.0127

[28] Pati S, Kavanagh JE, Bhatt SK, Wong AT, Noonan K, et al (2012) Reading level of medicaid renewal applications. Acad Pediatr 12: 297–301. 22682719 10.1016/j.acap.2012.04.008

[29] Sentell T (2012) Implications for reform: survey of California adults suggests low health literacy predicts likelihood of being uninsured. Health Aff (Millwood) 31: 1039–1048. 22566444 10.1377/hlthaff.2011.0954

[30] DeWalt DA, Hink A (2009) Health literacy and child health outcomes: a systematic review of the literature. Pediatrics 124 Suppl 3 S265–274. 19861480 10.1542/peds.2009-1162B

[31] Sanders LM, Federico S, Klass P, Abrams MA, Dreyer B (2009) Literacy and child health: a systematic review. Arch Pediatr Adolesc Med 163: 131–140. 19188645 10.1001/archpediatrics.2008.539

[32] Ferguson B (2008) Health literacy and health disparities: the role they play in maternal and child health. Nurs Womens Health 12: 286–298. 18715376 10.1111/j.1751-486X.2008.00343.x

[33] Sanders LM, Thompson VT, Wilkinson JD (2007) Caregiver health literacy and the use of child health services. Pediatrics 119: e86–92. 17200263 10.1542/peds.2005-1738

[34] Lee JY, Divaris K, Baker AD, Rozier RG, Lee SY, et al (2011) Oral health literacy levels among a low income population. J Public Health Dent 71: 152–160. 21774139 10.1111/j.1752-7325.2011.00244.x PMC3145966

[35] Kalton G (1983) Models in the practice of survey sampling. International Statistical Review 51: 175–188.

[36] Lee JY, Divaris K, Baker AD, Rozier RG, Vann WF Jr (2012) The relationship of oral health literacy and self-efficacy with oral health status and dental neglect. Am J Public Health 102: 923–929. 22021320 10.2105/AJPH.2011.300291 PMC3267012

[37] Divaris K, Lee JY, Baker AD, Vann WF Jr (2011) The relationship of oral health literacy with oral health-related quality of life in a multi-racial sample of low-income female caregivers. Health Qual Life Outcomes 9: 108. 22132898 10.1186/1477-7525-9-108 PMC3248838

[38] Vann WF Jr, Lee JY, Baker D, Divaris K (2010) Oral health literacy among female caregivers: impact on oral health outcomes in early childhood. J Dent Res 89: 1395–400. 20924067 10.1177/0022034510379601 PMC3123718

[39] Cooper WO, Arbogast PG, Hickson GB, Daugherty JR, Ray WA (2005) Gaps in enrollment from a Medicaid managed care program: effects on emergency department visits and hospitalizations for children with asthma. Med Care 43: 718–725. 15970788 10.1097/01.mlr.0000167174.05861.3d

[40] Weiss BD, Mays MZ, Martz W, Castro M, DeWalt DA, et al (2005) Quick assessment of literacy in primary care: the newest vital sign. Ann Fam Med 3: 514–522. 16338915 10.1370/afm.405 PMC1466931

[41] Welch VL, VanGeest JB, Caskey R (2011) Time, costs, and clinical utilization of screening for health literacy: a case study using the Newest Vital Sign (NVS) instrument. J Am Board Fam Med 24: 281–289. 21551400 10.3122/jabfm.2011.03.100212

[42] Osborn CY, Weiss BD, Davis TC, Skripkauskas S, Rodrigue C, et al (2007) Measuring adult literacy in health care: performance of the newest vital sign. Am J Health Behav 31 Suppl 1 S36–46. 17931135 10.5555/ajhb.2007.31.supp.S36

[43] Peixoto JL (1987) Hierarchical variable selection in polynomial regression models. The American Statistician 41: 311–313.

[44] Gardner MJ, Altman DG (1986) Confidence intervals rather than P values: estimation rather than hypothesis testing. Br Med J (Clin Res Ed) 292: 746–750. 10.1136/bmj.292.6522.746 PMC1339793 3082422

[45] Greenland S, Rothman KJ (2008) Introduction to stratified analysis. In: Rothman KJ, Greenland S, Lash TL. Modern epidemiology. New York: Lippincott, Williams and Wilkins. pp. 258–282.

[46] Fairbrother G, Madhavan G, Goudie A, Watring J, Sebastian RA, et al (2011) Reporting on continuity of coverage for children in Medicaid and CHIP: what states can learn from monitoring continuity and duration of coverage. Acad Pediatr 11: 318–325. 21764016 10.1016/j.acap.2011.05.004

[47] Yu J, Harman JS, Hall AG, Duncan RP (2011) Impact of Medicaid/SCHIP disenrollment on health care utilization and expenditures among children: a longitudinal analysis. Med Care Res Rev 68: 56–74. 20675347 10.1177/1077558710374620

[48] Sørensen K, Van den Broucke S, Fullam J, Doyle G, Pelikan J, et al (2012) Health literacy and public health: a systematic review and integration of definitions and models. BMC Public Health 12: 80. 22276600 10.1186/1471-2458-12-80 PMC3292515

[49] Berkman ND, Sheridan SL, Donahue KE, Halpern DJ, Crotty K (2011) Low health literacy and health outcomes: an updated systematic review. Ann Intern Med 155: 97–107. 21768583 10.7326/0003-4819-155-2-201107190-00005

[50] Sentell TL, Halpin HA (2006) Importance of adult literacy in understanding health disparities. J Gen Intern Med 21: 862–866. 16881948 10.1111/j.1525-1497.2006.00538.x PMC1831569

[51] Parker RM, Ratzan SC, Lurie N (2003) Health literacy: a policy challenge for advancing high-quality health care. Health Aff (Millwood) 22: 147–153. 12889762 10.1377/hlthaff.22.4.147

[52] Heinrich C (2012) Health literacy: the sixth vital sign. J Am Acad Nurse Pract 24: 218–223. 22486837 10.1111/j.1745-7599.2012.00698.x

[53] Sheridan SL, Halpern DJ, Viera AJ, Berkman ND, Donahue KE, et al (2011) Interventions for individuals with low health literacy: a systematic review. J Health Commun 16 Suppl 3 30–54. 21951242 10.1080/10810730.2011.604391

[54] Flores G, Abreu M, Brown V, Tomany-Korman SC (2005) How Medicaid and the State Children’s Health Insurance Program can do a better job of insuring uninsured children: the perspectives of parents of uninsured Latino children. Ambul Pediatr 5: 332–340. 16302834 10.1367/A04-067R2.1

[55] Ross DC, Hill IT (2003) Enrolling eligible children and keeping them enrolled. Future Child 13: 81–97. 14503455

[56] Pati S, Siewert E, Wong AT, Bhatt SK, Calixte RE, et al (2014) The Influence of Maternal Health Literacy and Child’s Age on Participation in Social Welfare Programs. Matern Child Health J 18: 1176–1189. 23990157 10.1007/s10995-013-1348-0 PMC3938979

[57] DeVoe JE, Krois L, Edlund T, Smith J, Carlson NE (2008) Uninsurance among children whose parents are losing Medicaid coverage: Results from a statewide survey of Oregon families. Health Serv Res 43: 401–418. 18199193 10.1111/j.1475-6773.2007.00764.x PMC2323132

[58] Yin HS, Johnson M, Mendelsohn AL, Abrams MA, Sanders LM, et al (2009) The health literacy of parents in the United States: a nationally representative study. Pediatrics 124: S289–298. 19861483 10.1542/peds.2009-1162E

[59] Chatterji P, Brooks-Gunn J (2004) WIC participation, breastfeeding practices, and well-child care among unmarried, low-income mothers. Am J Public Health 94: 1324–1327. 15284035 10.2105/ajph.94.8.1324 PMC1448447

[60] Lee JY, Rozier RG, Norton EC, Kotch JB, Vann WF Jr (2004) Effects of WIC participation on children’s use of oral health services. Am J Public Health 94: 772–777. 15117699 10.2105/ajph.94.5.772 PMC1448336

[61] Lillie-Blanton M, Hoffman C (2005) The role of health insurance coverage in reducing racial/ethnic disparities in health care. Health Aff (Millwood) 24: 398–408. 15757923 10.1377/hlthaff.24.2.398

[62] Chassin MR, Galvin RW (1998) The urgent need to improve health care quality. Institute of Medicine National Roundtable on Health Care Quality. JAMA 280: 1000–1005. 9749483 10.1001/jama.280.11.1000

[63] Congressional Budget Office Bulletin (2012) “Updated Estimates for the Insurance Coverage Provisions of the Affordable Care Act”. Available: http://cbo.gov/sites/default/files/cbofiles/attachments/03-13-Coverage%20Estimates.pdf. Accessed 2014 Sept 19.

[64] Baicker K, Congdon WJ, Mullainathan S (2012) Health insurance coverage and take-up: lessons from behavioral economics. Milbank Q 90: 107–134. 22428694 10.1111/j.1468-0009.2011.00656.x PMC3385021

[65] Sommers BD, Tomasi MR, Swartz K, Epstein AM (2012) Reasons for the wide variation in Medicaid participation rates among states hold lessons for coverage expansion in 2014. Health Aff (Millwood) 31: 909–919. 22566429 10.1377/hlthaff.2011.0977

[66] Kronebusch K, Elbel B (2004) Simplifying children’s Medicaid and SCHIP. Health Aff (Millwood) 23: 233–246. 15160822 10.1377/hlthaff.23.3.233

